# Pneumonia in Children

**Published:** 1907-08

**Authors:** 


### PNEUMONIA IN CHILDREN.

Murry states that in the case of young children suffering from acute disease of the lungs, it is of more importance to keep an accurate record of the respiration rate than of the pulse rate. It is a measure of the defective aeration of the blood, and reminds us that a prolonged and considerable rise in the respiration rate may be followed by a rapidly culminating exhaustion, matters of great importance in prognosis and treatment. In the early stages of acute pneumonia in children, apart from rickets, the extent and duration of the disease are the chief factors in eccelerating the rate of breathing. The writer disbelieves in the current classification of pneumonia, in children into lobar pneumonia and bronchopneumonia, holding that it is inaccurate and of no clinical utility. The symptoms and signs are so variable and are met with in such different combinations, that classification is almost impossible. To him bronchopneumonia simply means a pneumonia that in spreading seems to follow the distribution of the bronchial tubes, and lobar pneumonia one that spreads in a fairly even advancing line; in this sense only is bronchopneumonia a commoner disease of childhood than lobar-pneumonia. Each sign and symptom should be estimated individually as to its influence on prognosis and treatment, rather than all being lumped together in an arbitrary, hypothetical group. From a study of fifty-four cases of acute pneumonia in children, the following was learned: The more definitely localized the pneumonia, the more sudden the onset. Vomiting is the most constant of the early symptoms, while convulsions are extremely rare as an initial symptom. Pain, an important and significant symptom in the adult, is rarely noteworthy in childhood. The feature most difficult to classify was the fever, it being impossible to group the cases into "continuous" and "remittent." Empyema and pericarditis are most exceptional conplications in children. Relapses are always due to direct extension either from the original focus or from some other patch of consolidation. The two most important factors in forming a prognosis are age and nutrition. Most of the cases under one year of age die. Rickets is a very serious factor, especially in prolonged cases. The more sudden the onset and the more localized the consolidation, the better are the patient's chances. Because of the importance of wasting and of continued vomiting, special attention should be paid to the diet. The writer does not employ ice bags or venesection. A free supply of fresh air is of prime importance. Drugs have little influence over the course of pneumonia in childhood, except as stimulants to the circulation or respiration.—British Medical Journal, June 8th, 1907.

